# Changes in noise levels in the city of Madrid during COVID-19 lockdown in 2020a)

**DOI:** 10.1121/10.0002008

**Published:** 2020-09-25

**Authors:** César Asensio, Ignacio Pavón, Guillermo de Arcas

**Affiliations:** Instrumentation and Applied Acoustics Research Group, Universidad Politécnica de Madrid, Madrid, c/ Mercator 3, 28031 Madrid, Spain

## Abstract

The lockdown that Madrid has suffered during the months of March to June 2020 to try to control and minimize the spread of COVID-19 has significantly altered the acoustic environment of the city. The absence of vehicles and people on the streets has led to a noise reduction captured by the monitoring network of the City of Madrid. In this article, an analysis has been carried out to describe the reduction in noise pollution that has occurred and to analyze the changes in the temporal patterns of noise, which are strongly correlated with the adaptation of the population's activity and behavior to the new circumstances. The reduction in the sound level ranged from 4 to 6 dBA for the indicators L_d_, L_e_, and L_n_, and this is connected to a significant variation in the daily time patterns, especially during weekends, when the activity started earlier in the morning and lasted longer at midday, decreasing significantly in the afternoon.

## I. INTRODUCTION

The year 2020 will be remembered as the year of the coronavirus disease pandemic (COVID-19), which has caused thousands of deaths all over the world. The main strategy that most countries have applied to combat the spread of the pandemic and its effects has been to establish measures for restricting the mobility of people by means of a lockdown, which has kept large sectors of the population confined to their homes. More than 3.9 × 10^9^ people, living in 90 different countries, have been subjected to confinement as a measure to maintain social distance (Sandford, 2020; Aktay *et al.*, 2020). Commercial flights have been restricted, and flights not dedicated to medical supplies or essential commodities have been affected in many countries (Olive *et al.*, 2020). Ground transportation has also been severely limited, as a significant percentage of the population has not been allowed to access their jobs or has had to work remotely (Tardivo *et al.*, 2020).

In Spain, Madrid has been one of the regions where the pandemic has hit hardest and earliest, and because of this, the lockdown has been longer and more severe than in other regions. Lockdown in Madrid developed in various stages, with measures that have evolved over time. On March 12, the regional government suspended classes at all educational levels, starting the “No Class” (NC) stage. On March 14, the national government declared a state of emergency. Until March 28, this “Stay Home” stage (SH) had schools closed, events suspended, and non-essential shops closed. Tele-work was mandatory for every job that allowed this option. Everyone had to stay home, except for essential work or purchases. No outdoor walks or sports were allowed. Province borders were closed. On March 29, the lockdown got stricter, since all non-essential movement was banned (“Only Essential,” OE stage). This situation lasted until April 13, when SH stage returned for 14 more days, lasting until April 26. From April 27, the de-escalation process started, with a first stage “D0”. With major restrictions, the practice of outdoor sports and walks was allowed again. Tele-work was still mandatory where possible, and province borders were closed. On May 25, the new de-escalation stage (D1) relaxed the lockdown measures, allowing many workers to return to their jobs, removing time restrictions on outdoor movements, and laying the groundwork for an economic recovery (Table I).

**TABLE I.** Stages of lockdown in Madrid.

Stage	Starting	Ending	Description
NC (No class)	March 12	March 13	Schools suspended; Tele-work suggested
SH (Stay home)	March 14	March 28	School closed; Events suspended; Non-essential shops closed; Province borders closed; Tele-work unless justified; No walks or sports outdoors
OE (Only essential)	March 29	April 12	School closed; Events suspended; Non-essential shops closed; Province borders closed; No walks or sports outdoors; Non-essential movement banned
SH (Stay home)	April 13	April 26	School closed; Events suspended; Non-essential shops closed; Province borders closed; Tele-work unless justified; No walks or sports outdoors
D0 (De-escalation 0)	April 27	May 24	School closed; Events suspended; Province borders closed; Tele-work unless justified; Walks and sports outdoors allowed (major restrictions)
D1 (De-escalation 1)	May 25	June 7	School closed; Events suspended; Province borders closed; Tele-work suggested; Walks and sports outdoors allowed (minor restrictions); Economic restarting measures;

Together with the tremendous and regrettable consequences of the pandemic, the lockdown that has been implemented to mitigate against it has changed the environment significantly, especially in large cities such as Madrid (Ball, 2020; Venter *et al.*, 2020; Institute of Acoustics, 2020; Bui and Badger, 2020; Zambrano-Monserrate *et al.*, 2020; Baldasano, 2020; Eurocities, 2020).

The absence of vehicles and people on the streets has generated an unprecedented scenario, which has reduced the noise pollution in the city, altering its soundscape (Peinado and Clemente, 2020). In this paper, we aim to describe this new acoustic situation, taking advantage of the extensive noise monitoring network installed in Madrid and following the guidelines presented in Asensio *et al.* (2020). Unlike other initiatives, which focus more on the qualitative and perceptual changes that have occurred in relation to the soundscape (Institute of Acoustics, 2020; Locate Your Sound, 2020; Cities & Memory, 2020), or how this change has been perceived or has affected people, in this article we focus on quantitative data, measured directly by the monitoring network, and try to describe the reduction in purely objective terms that are related to the acoustic energy reduction. This is an approach that has also been carried out in other studies (Acoucite, 2020; Challéat *et al.*, 2020; Mandal and Pal, 2020; Bruitparif, 2020; Eurocities, 2020; Sakagami, 2020; Ajuntament de Barcelona, 2020; Aletta *et al.*, 2020), in which we intend to go deeper by analyzing not only the basic acoustic indicators but also the global trends and acoustic patterns throughout a day, and by making a distinction between working days and weekends.

## II. THE NOISE MONITORING NETWORK

**FIG. 1.**
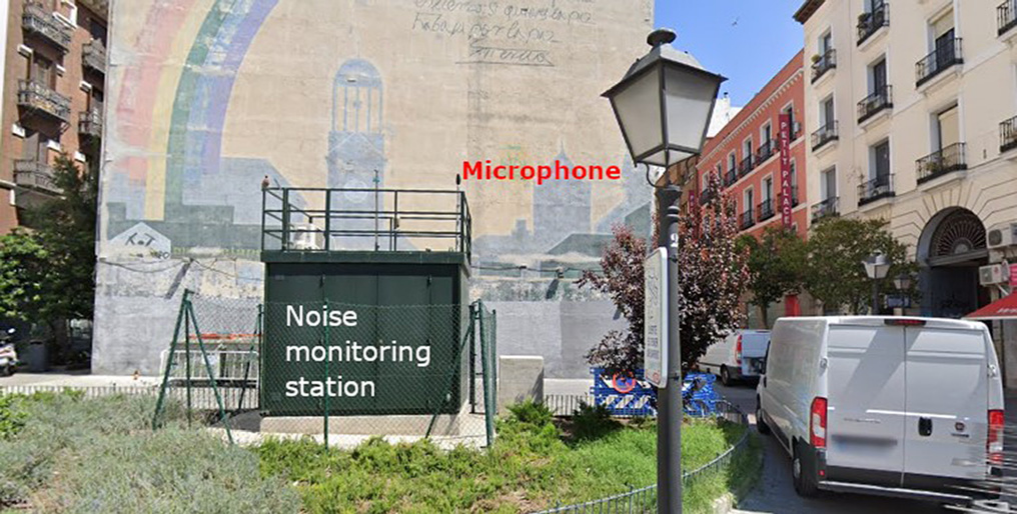
(Color online) Noise monitoring station (Plaza del Carmen).

The noise monitoring network in Madrid has 31 monitoring terminals placed throughout the city. These are Bruel & Kjaer class 1 sound level meters with an outdoor microphone, which are calibrated every year in accordance with the legal metrology requirements in force in Spain. They are installed on the roof of an environmental conditions measurement booth in compliance with the basic installation requirements specified in the ISO 1996 standard, as shown in Fig. 1 (ISO, 2017).

**FIG. 2.**
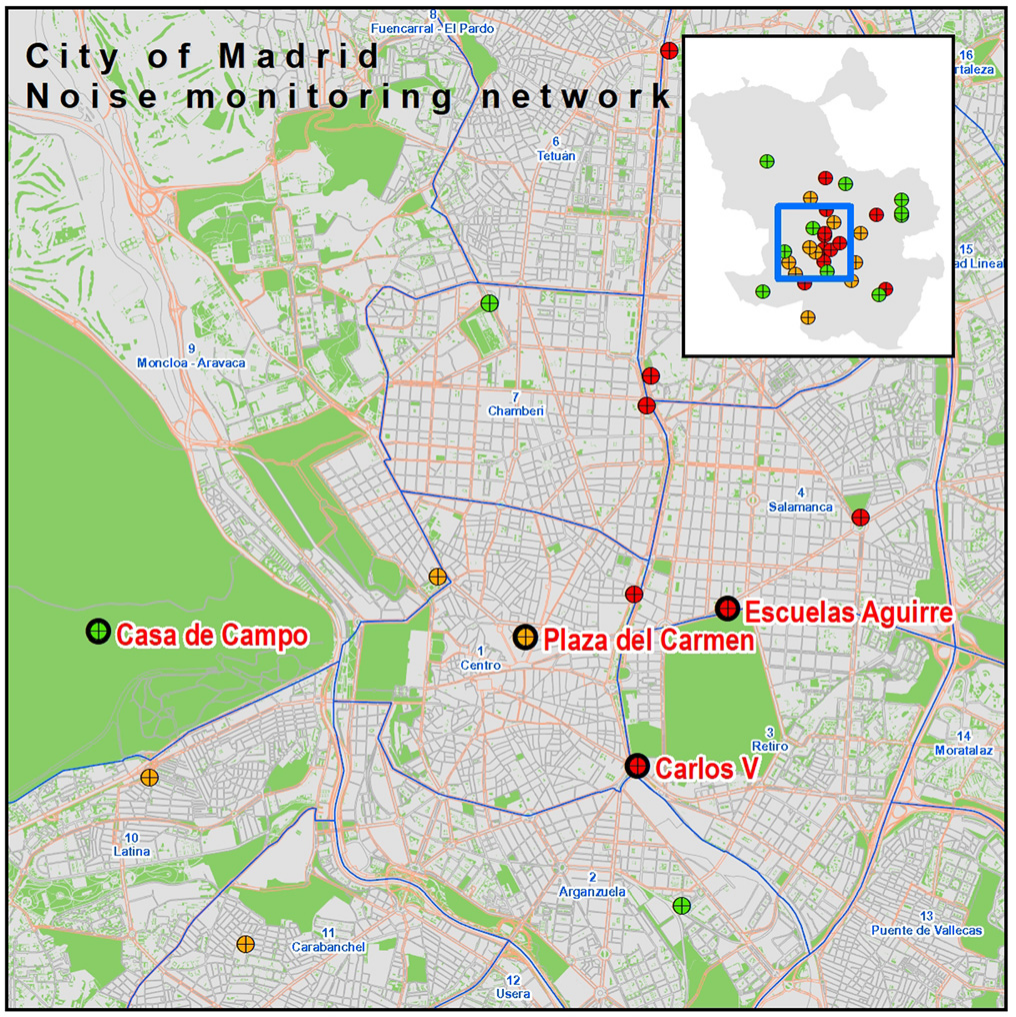
(Color online) Noise monitoring location in Madrid's network. Zoomed and highlighted Carlos V, Plaza del Carmen, Escuelas Aguirre and Casa de Campo.

This article aims to provide general information on the effects that the lockdown has had on noise pollution in the city as a whole, without focusing on any specific location. Applying a clustering and classification process similar to that described by Aletta and colleagues (2020), the measurement locations have been classified according to three categories:

• Traffic-dominated areas (10 locations, red in Fig. 2), located near major roads in the city.
• Active areas (10 locations, orange in Fig. 2), areas in the city center where road traffic is also present, but commercial, tourism, and leisure noise have an important role too.
• Quiet areas (11 locations, green in Fig. 2), which include quietest monitoring locations, mainly in green areas, parks, and calm residential areas on the outskirts of the city.

To show the detail of some of the analyses carried out, five locations have been selected, which we consider to be significant. In Fig. 2 we zoom in and highlight four of the selected terminals. Carlos V is located next to the Atocha train station, which has very intense traffic flow in its surroundings (traffic dominated area). The Aguirre Schools location is very close to the Retiro Park (the largest green area in the city center), next to two important roads, Alcalá and Odonnel streets (traffic dominated area). Also very central is the Plaza del Carmen, which is in an area with less traffic, but is an important commercial and tourist activity (active area). We also chose a location, further from the center of the city, in the Casa de Campo, an important green area on the outskirts of the city (quiet area). Outside the map, we selected the fifth reference point in Sanchinarro, as it is a more recently built area with a predominantly residential use, located in the northern expansion of the city (quiet area).

## III. ANALYSIS AND RESULTS

### A. Time series along the lockdown

In these five locations, we have analyzed in detail the time history of the noise levels throughout 2020. To do this, we have used the equivalent continuous sound level data averaged over 1 h (L_Aeq,1h_), provided by the Madrid City Council between the months of February and June.

**FIG. 3.**
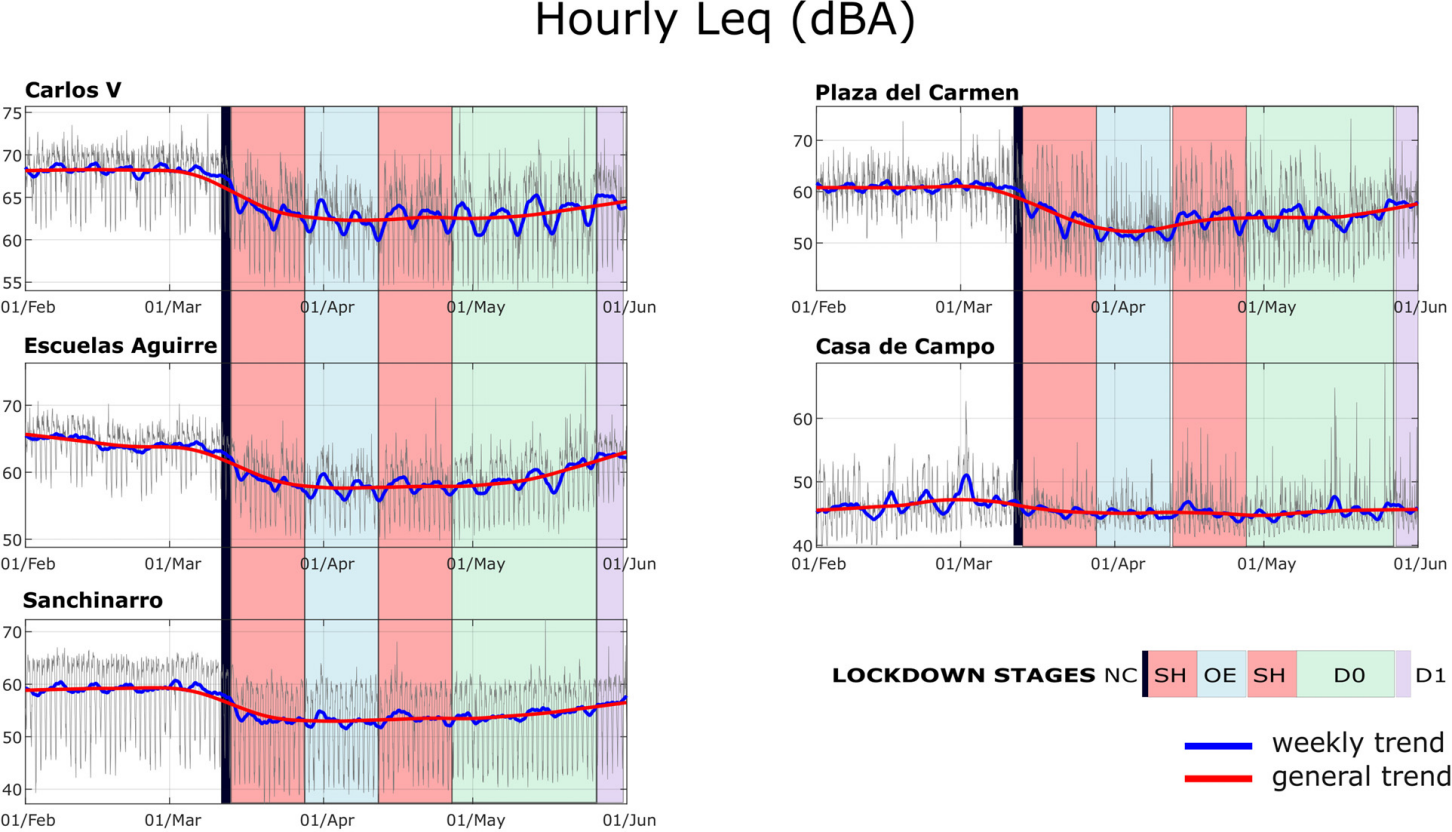
(Color online) Hourly noise level time series in five selected locations.

Figure 3 shows the time series of this descriptor over time, with the strong variations that occur throughout the day. To facilitate the analysis, two levels of smoothing have been superimposed on the graph. The first one shows a certain ripple that allows us to visualize the reduction of the sound level that most of the points experience during the weekend. The second shows a long-term trend at these five measurement points. We can clearly observe that the dynamics of the hourly measurements are quite important at all locations, showing significant variations between the levels measured at peak times and those measured at the quietest moments of the night. This variation is more pronounced during the lockdown, because the sound level reduction is a few decibels greater at night. To a similar order of magnitude, the decrease is greater during the weekends than on weekdays, which translates into a ripple in the smoothed graph that is very evident during the lockdown and almost unnoticeable before March.

**FIG. 4.**
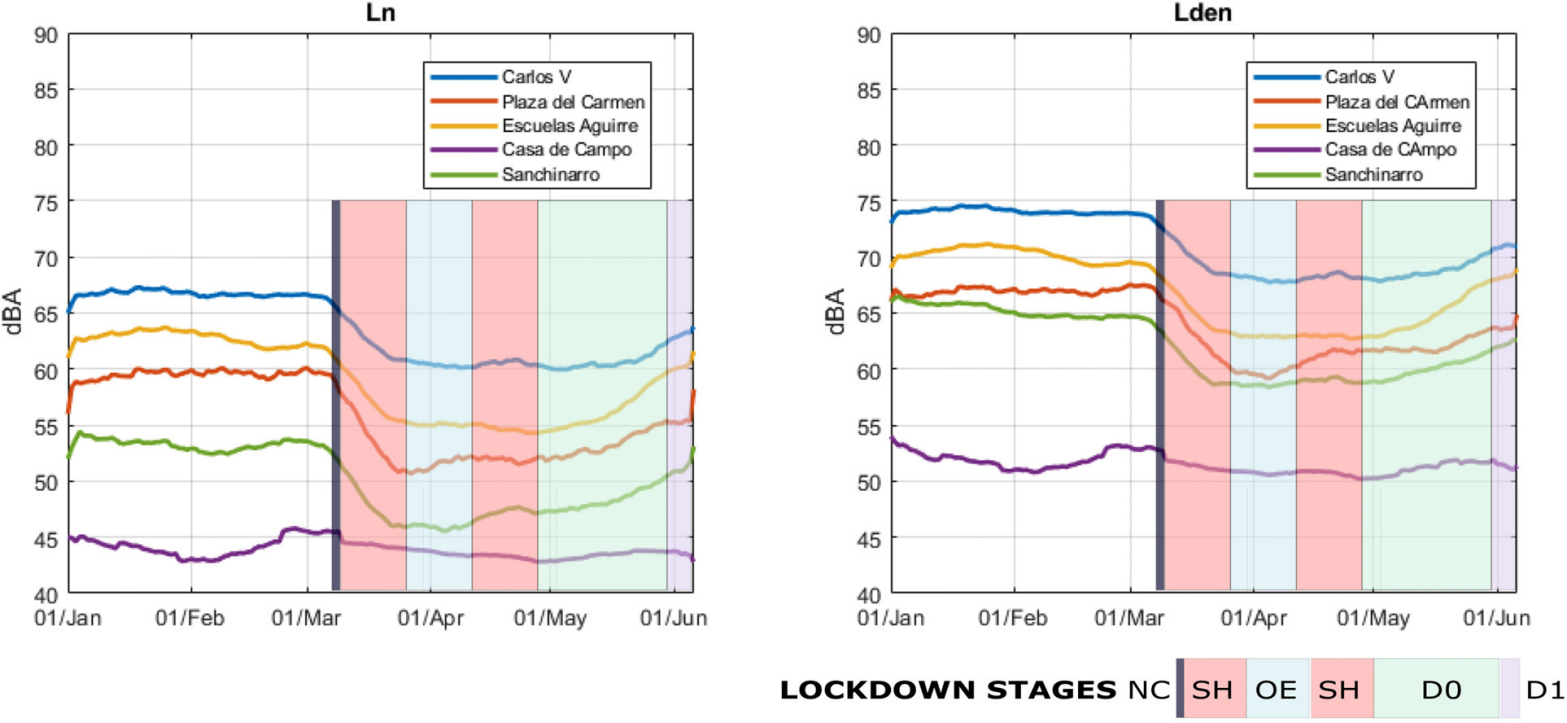
(Color online) L_den_ and L_n_ time series in five selected locations.

When we analyze the time series of the indicators defined in the European Environmental Noise Directive (European Parliament, 2002), L_den_ and L_n_ smoothed out in Fig. 4. We realize that, as in usual situations, there is a strong correlation between the two indicators, because it is the traffic noise, with its time patterns, that mostly determines their behavior. It can be found that the drop in the sound level was very fast with the start of the lockdown. Although it took several days for the sound level to reach its minimum, it had already achieved this by the end of April, when the hardest stage of the lockdown came into effect (OE), and therefore this new and more severe stage did not, in general terms, bring any further reduction. On the contrary, the end of the OE phase turned out to be a turning point and the beginning of an increase in the sound level, especially in the areas that are predominantly residential (in our selection, Plaza del Carmen and Sanchinarro). Gradually, the sound levels started to increase slightly, which could indicate a higher level of activity in the streets. In the areas closest to traffic arteries, however, sound levels remained stable for one more month, until the end of April. At every location, the start of de-escalation (D0) meant the beginning of the recovery of the acoustic environment in the city, and the entry into phase D1 of this de-escalation had, once again, a very notable effect on the recovery of the noise levels.

It should be noted that Casa de Campo registered a totally anomalous behavior to the rest of the locations, which will be addressed specifically in Sec. III C.

Table II allows us to quantify the reduction for each of these five reference locations.

**TABLE II.** L_den_ and L_n_ indicators in five reference locations.

Locations	Carlos V		Pza. del Carmen		Sanchinarro		Escuelas Aguirre		Casa de campo	
Indicators	Before	Lckdwn	Before	Lckdwn	Before	Lckdwn	Before	Lckdwn	Before	Lckdwn
% days exceeding L_den_ = 65 dBA	100	100	99	1	52	0	100	29	0	0
% days exceeding L_n_ = 55 dBA	100	100	100	12	10	0	100	52	1	0
Average L_Aeq,1 h_ during rush hour (dBA) - Weekdays	71	67	67	62	65	55	67	58	50	48
Average L_Aeq,1 h_ during day period off-peak hour (dBA) - Weekdays	70	64	60	53	60	52	66	57	46	46
Average L_Aeq,1 h_ during rush hour (dBA) - Weekend	70	72	62	51	65	60	66	59	53	45
Average L_Aeq,1 h_ during day period off-peak hour (dBA) - Weekend	67	60	58	51	59	52	62	56	47	44
Average L_den_ (dBA)	74	69	67	62	65	60	70	65	53	51
Average L_n_ (dBA)	67	61	60	53	54	48	63	57	46	44

### B. Overall noise reduction

**FIG. 5.**
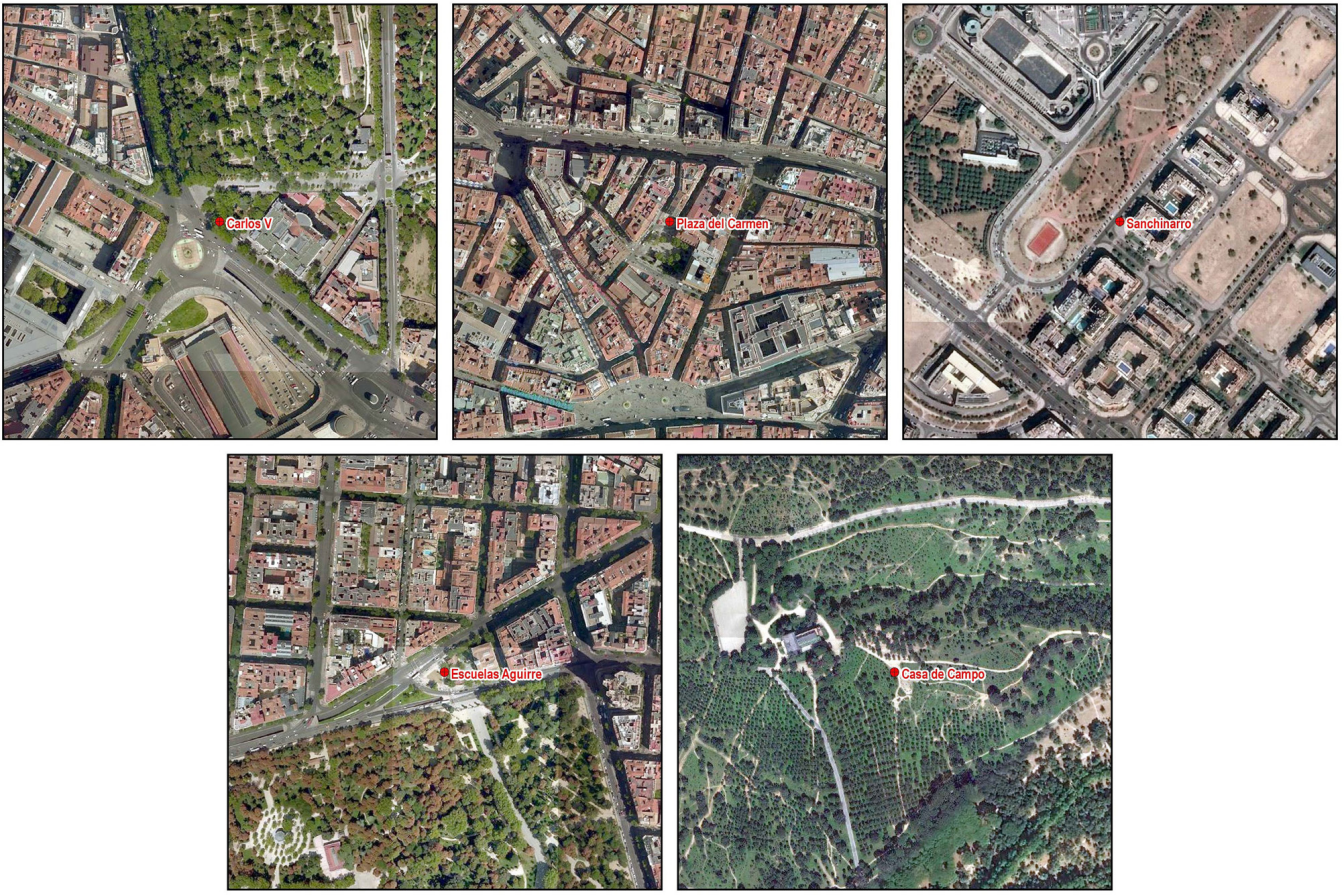
(Color online) Aerial photographs of the selected locations.

Section III B will present statistics describing the noise reduction achieved throughout the city, based on data from the 31 monitoring stations. In view of the previous analyses, we have decided that it is appropriate to redefine the stages for the present analysis: “lockdown” (from March 16 to May 31) and “before lockdown” (from February 1 to March 10), and we have analyzed separately the three different area classes that have been previously described. Table III shows the mean noise reduction that the lockdown has produced for every indicator and every reference location in Fig. 5.

**TABLE III.** Average noise reduction during weekdays and weekends.

	Noise reduction (dBA)		
	L_d_	L_e_	L_n_
Weekday / Traffic dominated area	4.0	3.9	4.6
Weekday / Active area	4.4	4.0	4.1
Weekday / Quiet area	4.2	4.4	4.1
Weekend / Traffic dominated area	5.6	5.7	7.4
Weekend / Active area	4.5	3.9	6.3
Weekend / Quiet area	5.2	4.9	5.6

**FIG. 6.**
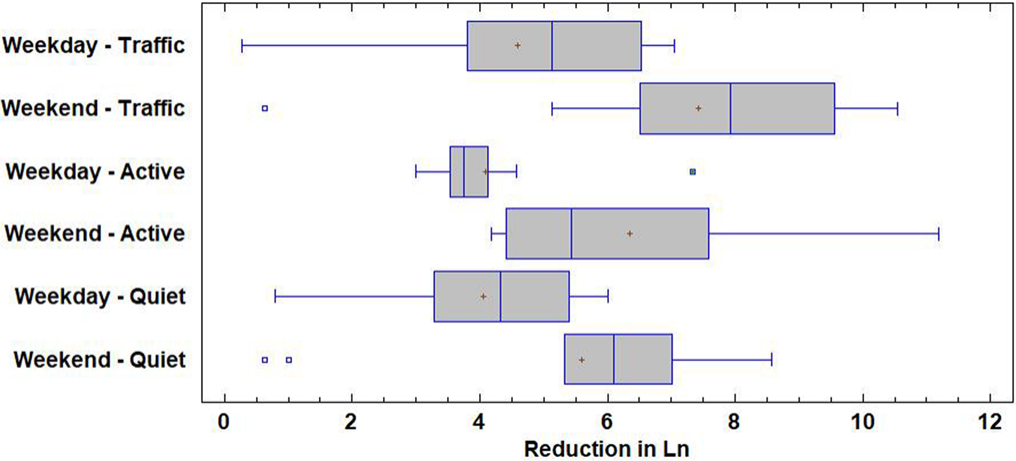
(Color online) Box plot for weekday and weekend noise reduction (indicator L_n_).

The average reduction of the sound level during working days is close to 4 dB in the day, evening, and night indicators, in every area type. The day and night indicators experience a greater reduction during the weekends than during the weekdays. In Fig. 6, we observe the phenomenon for the night indicator, where the effect is more noticeable. We observe how in all three types of areas the sound level is reduced more during the weekend, due to the absence of leisure and commercial activity. This reduction is more visible in areas dominated by traffic noise, where the mean reduction reaches 7.4 dB, and the quartiles Q1 and Q3 reach 6.5 and 9.5 dBA, respectively. However, the highest reduction (11.2 dB) is observed in the indicator L_n_ in Plaza del Carmen, which is classified as an active area.

It is noteworthy that there is a location where, unexpectedly, the night indicator has increased during shutdown. This is in “Campo de las Naciones,” an office area with scarce night traffic in regular times, where, during the confinement, a field hospital was set up to provide assistance to patients with COVID. Apart from this location, the reduction in the sound level has been consistent during the weekend night period. In fact, there are only three locations where this reduction is less than 1 dB: one of them is in a traffic dominated area (Plaza Castilla) and two others are in quiet areas (Casa de Campo and Mendez Alvaro). The location of Casa de Campo also presents a very low reduction in all the indicators during working days, which is noticeable only in the weekend L_d_ and L_e_ indicators.

### C. Changes in time patterns

In addition to the changes in noise over time, and the reduction observed in the main indicators, in this research we have attempted to analyze the influence that lockdown has had on the modification of time patterns throughout the day, since the noise levels are highly correlated to the activity in the city. We have used the measurements of equivalent continuous sound pressure levels in 1 h intervals (L_Aeq,1h_), measured at every station.

**FIG. 7.**
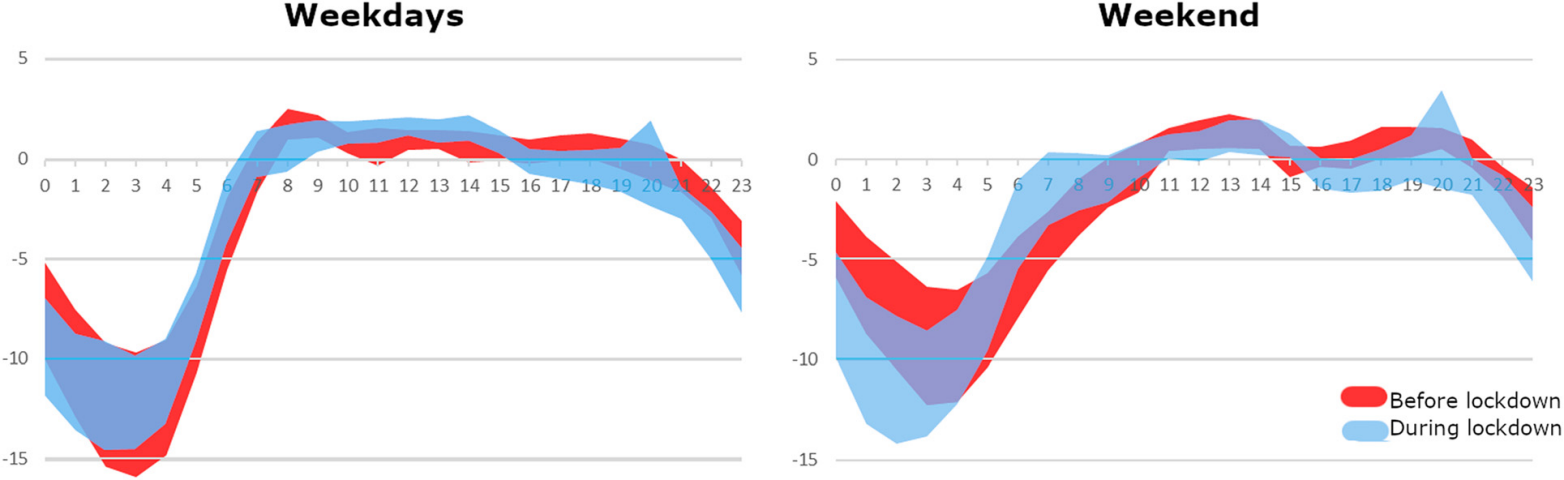
(Color online) Tolerance range for the changes in daily noise patterns during lockdown.

In each of the stages (“lockdown” and “before lockdown”), and for each location, the average time evolution of the sound level on a working day and on a weekend day has been calculated. To make the resulting patterns comparable from one location to another, the value of the 24-h equivalent sound level (L_Aeq,24h_) at that location has been subtracted from each hourly value (L_Aeq,1h_). In this way, we observe that most locations show a very similar daily pattern. Figure 7 shows a range that characterizes the average value of all locations and a 95% tolerance interval, during the lockdown and before the lockdown, for a working day and a weekend day.

From the results shown in Fig. 7, we can clearly identify changes in the time patterns of noise throughout the day. Before the lockdown, weekdays were quite busy from about 7 a.m. to about 9 p.m., with peak noise levels between 8 and 9 a.m. With confinement, a peak hour is not so clearly discerned, but a reduction in noise activity is clearly observed from 2 to 3 p.m. onward. A noise peak is observed at 8 p.m., which coincided with the applause with which the people of Madrid thanked and paid tribute to the healthcare and other essential workers. From 9 p.m. on, noise levels strongly decrease, which seems to reflect the reduction over a couple of hours the activity in the streets during the lockdown (compared to a regular situation).

On weekends, the hourly pattern of noise is different. Before the lockdown, there were two clearly noisier periods, separated by lunchtime between approximately 2 and 5 p.m. During the lockdown, two noisy periods are still observed. Unlike before the lockdown, the morning period reaches higher sound levels and has a longer time span than the afternoon period. It begins between 7 and 11 a.m. (when it was between 9 and 11 a.m. before the lockdown), and lasts until almost 4 p.m., when it ended an hour earlier before the confinement. Although there was also a certain amount of noise in the afternoon, it does not reach morning levels, and it ends at about 9 p.m., an hour earlier than before the lockdown. Also on weekends, 8 p.m. is the noisiest time of the day, because of the applause.

**FIG. 8.**
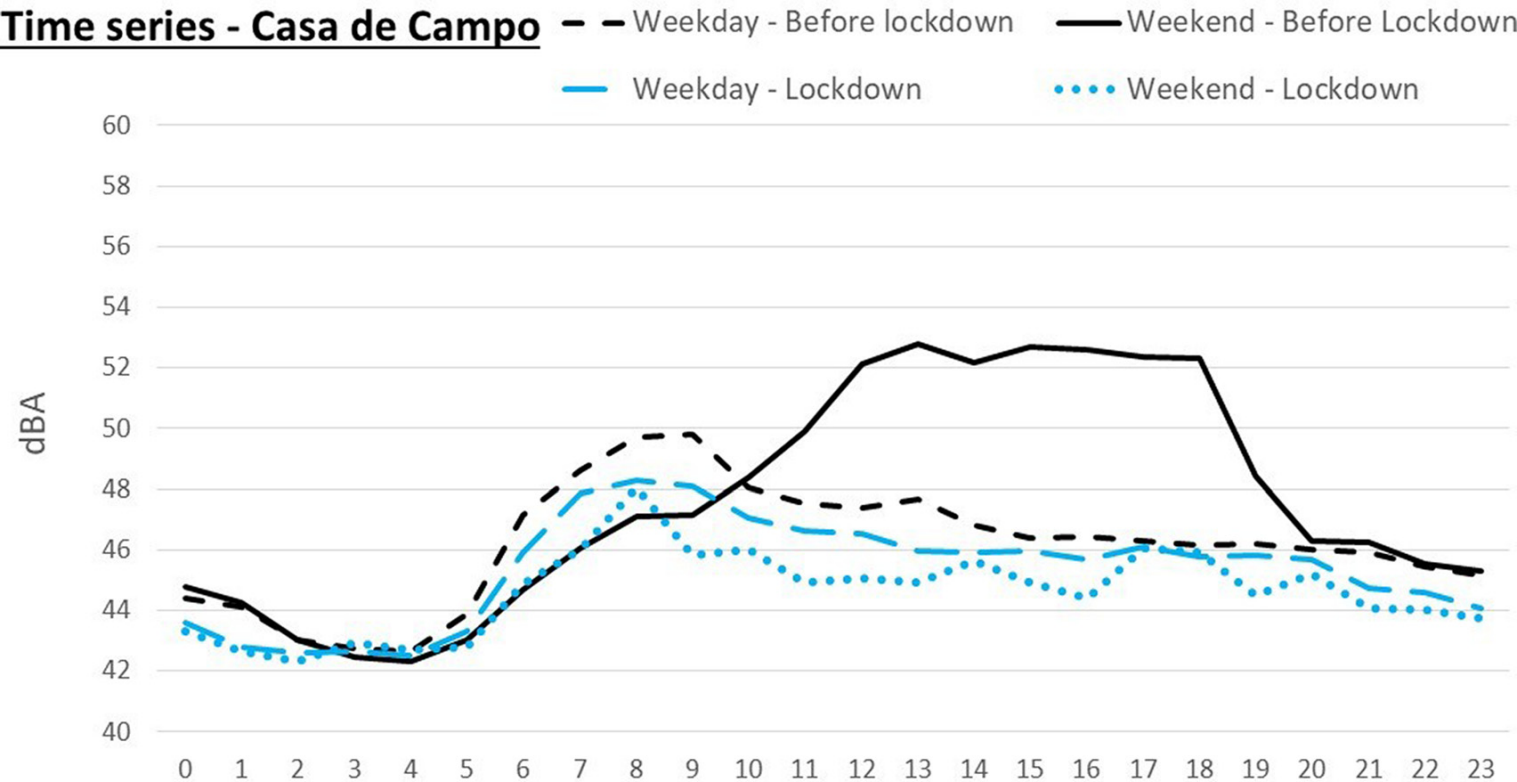
(Color online) Time series of the sound level during an average day in Casa de Campo.

We must make special mention of the reference point located in the Casa de Campo. This is a large green area where people in Madrid walk or play sports, and it also has an amusement park and a zoo. Due to its use, clearly different from the other locations, the activity in this area, and the associated traffic, has a different time pattern. This area remains relatively quiet during the weekdays, with an extremely low volume of traffic, compared to the weekends, when the influx of visitors is significant, especially in the proximity of the zoo and the amusement park. In Fig. 8, we can note that the daily time pattern during the lockdown shows little variation between weekdays and weekends, since during the confinement all the activity remained closed. Furthermore, these time noise patterns were quite similar to a weekday before the confinement. We must focus on the time pattern of noise in the area during the weekends to note that, in fact, the reduction in sound level has reached here. Since the 4–6 dBA noise reduction occurs only on weekends, and only for a limited number of hours, it has an small overall effect that is different from what we have observed in the rest of the locations in the network.

## IV. CONCLUSION

The results of the study show a significant reduction in noise levels in the city of Madrid during confinement. This reduction affects the whole city, although it becomes more visible in the locations closer to the main traffic arteries (traffic dominated areas). Confinement has also affected the activity, behavior, and attitudes of citizens in the streets, so a reduction is also observed in those locations less affected by traffic noise and more affected by noise from leisure, tourism, and commercial activities (active areas).

Lockdown has not only reduced the intensity of noise but has also significantly changed its time patterns. The daily activity seems to start earlier, especially during weekends, but above all, it ends much earlier, as the lockdown has severely affected people's leisure time and outdoor activity.

In quantitative terms, the average reduction obtained in the city is between 4 and 5 dBA for indicators L_d_, L_e_, and L_n_ on working days, and even exceeds 6 dBA during the weekend night period. This can be explained by the lack of activity linked to nightlife activity during the weekends. Obviously, in some specific areas (such as Plaza del Carmen, in this sample), part of this reduction is due to the reduced presence of people in the streets, bars, and terraces enjoying nightlife and commercial activities (active areas). But above all, the generalized reduction is due to the absence of the usual night-time traffic on the streets during the weekend, a situation also linked to the absence of nightlife activity during the lockdown. This shows us how road traffic also has an important weight linked to nightlife noise, even though it is not usually the main concern for residents of nightlife areas. The absence of traffic is perceived in traffic dominated and active areas, but also in the locations placed in quiet areas.

It has been observed that the dynamics of noise, the difference between maximum and minimum levels (rush and off-peak hours), has increased during lockdown. Noise has decreased during peak hours, but more so during off-peak hours. This trend is also beginning to show up in weekly analysis. Although the whole week is quieter during lockdown, the reduction is more noticeable on weekends.

Although these conclusions can be generalized within the urban area, a big outlier has been observed, which corresponds to the Casa de Campo, whose characteristic activity is very different from the urban area, and is therefore less affected by the changes caused by lockdown.

The reduction of noise levels in Madrid seems to be quite consistent with those reported in other big cities (Barcelona, Paris, Lyon, New York) and clearly shows us the influence of the traffic on the sound environment of the city. We observe that the reduction achieved is lower than what would have been expected with the huge reduction in traffic volumes (approx. 85% in Madrid), which is due, on the one hand, to the higher speed of the remaining vehicles, and, on the other, to the greater distance covered by the sound levels generated on the major roads in the absence of the noise caused by local traffic on less busy streets.
